# Species origin of exogenous transcription factors affects the activation of endogenous pluripotency markers and signaling pathways of porcine induced pluripotent stem cells

**DOI:** 10.3389/fcell.2023.1196273

**Published:** 2023-04-21

**Authors:** Meng Zhou, Manling Zhang, Tianxu Guo, Lihua Zhao, Xiyun Guo, Zhibao Yin, Linxin Cheng, Han Liu, Lixia Zhao, Xihe Li, Rongfeng Li

**Affiliations:** ^1^ Jiangsu Key Laboratory of Xenotransplantation, Nanjing Medical University, Nanjing, China; ^2^ Key Laboratory of Targeted Intervention of Cardiovascular Disease, Collaborative Innovation Center for Cardiovascular Disease Translational Medicine, Nanjing Medical University, Nanjing, China; ^3^ The State Key Laboratory of Reproductive Regulation and Breeding of Grassland Livestock, College of Life Science, Inner Mongolia University, Hohhot, Inner Mongolia, China

**Keywords:** species, exogenous transcription factors, porcine induced pluripotent stem cells, endogenous pluripotency marker, activation

## Abstract

The incomplete silencing of exogenous transcription factors (TFs) and the lack of endogenous counterpart activation hampers the application of porcine induced pluripotent stem cells (piPSCs). We used porcine, bovine and murine TFs to reprogram porcine fetal fibroblasts. Porcine TFs-derived piPSCs (ppiPSCs) showed the highest levels of endogenous pluripotency markers activation, were able to differentiate into three germ layers and primordial germ cell-like cells (PGCLCs) and integrated into neural ectoderm of E7.5 mouse embryos *in vitro*. The bovine TFs derived piPSCs (bpiPSCs) expressed endogenous pluripotency markers higher than murine TFs derived piPSCs (mpiPSCs), but both had limited differentiation ability *in vitro* and depended on continuous expression of exogenous TFs for the maintenance. RNA sequencing confirmed ppiPSCs had distinct global transcriptional profiling, upregulated Hippo, PI3K-Akt, MAPK and relevant pluripotency signaling pathways as porcine blastocyst inner cell mass and expressed PGC early related genes. In addition, a positive and a negative correlation between exogenous and endogenous TFs’ expression level were observed in ppiPSCs and bpiPSCs lines, respectively. The TFs’ protein structures in pig were more similar to cattle than to mouse. In conclusion, the species affinity of the exogenous TFs is a key element, and the own species origin of TFs is optimal for iPSCs generation and application.

## 1 Introduction

Pigs are regarded as the ideal experimental animals for disease models, xenotransplantation and regenerative medicine research because of their similarities to humans in terms of metabolic physiology and organ size (Duran-Struuck et al., 2015; Stricker-Krongrad et al., 2016; Walters et al., 2017). The current pig models were mostly produced by gene-editing fetal fibroblasts followed by somatic cell nuclear transfer (SCNT) thus the editing of multiple genes usually needs several rounds of cell transfection and SCNT. The establishment of the appropriate porcine pluripotent stem cells (PSCs), either embryonic stem cells (ESCs) or induced pluripotent stem cells (iPSCs), will be helpful for this problem resolution (Robinton and Daley, 2012; Secher et al., 2016). Many efforts have been made to establish porcine ESCs (Hou et al., 2016; Gao et al., 2019; Zhang et al., 2019) *via* seeding the blastocyst inner cell mass directly *in vitro* and porcine iPSCs *via* reprograming the fibroblasts (Ezashi et al., 2009; Du et al., 2015; Choi et al., 2016; Li et al., 2018; Ma et al., 2018), however, the porcine PSCs with germline transmission competency have not been achieved yet.

The original studies on the generation of porcine iPSCs (piPSCs) were first reported in 2009 (Esteban et al., 2009; Ezashi et al., 2009; Wu et al., 2009). The piPSCs were generated from porcine somatic cells by exogenously expressing human or mouse origins of 4 TFs, *OCT4*, *SOX2*, *KLF4* and *CMYC* (OSKM), or 6 TFs, OSKM plus *NANOG* and *LIN28* (OSKMNL). These initially reported piPSCs morphologically resembled the conventional human ESCs. The subsequent attempts in several groups resulted in the generation of naïve-like state of piPSCs similar to mouse ESCs in some characters, however, these naïve-like piPSCs depended on the continuous expression of the exogenous TFs for their proliferation and self-renewal (Fujishiro et al., 2013; Gu et al., 2014), which is largely attributed to the insufficient activation of the endogenous signature pluripotency markers, such as *OCT4* and *KLF4*. To improve the nuclear reprogram in piPSCs, researchers investigated the effects of the initial cell types (Wu et al., 2009; West et al., 2011; Zhang et al., 2014; Setthawong et al., 2019; Xu et al., 2019; Recchia et al., 2022), the different TFs in different combination (Ji et al., 2013; Wang et al., 2013; Mao et al., 2017; Shi et al., 2020; Chakritbudsabong et al., 2021) and the culture conditions with discrete small molecular inhibitors (Fujishiro et al., 2013; Choi et al., 2016; Yuan et al., 2019; Pieri et al., 2022). In these studies, murine and human TFs still were most commonly used for cell reprogramming. Esteban et al. (Esteban et al., 2009) did not find the obvious differences between the two kinds of piPSCs regarding the morphology and pluripotency. Recchia et al. (Recchia et al., 2022) and Pieri et al. (Pieri et al., 2022) found the human TFs derived piPSCs underwent spontaneous differentiation and could not maintain typical colonies after passaging. Ji et al. (Ji et al., 2013) first used porcine and monkey TFs and found only three piPSCs lines containing monkey TFs and one piPSCs line containing porcine TFs exhibited silencing or partial silencing of exogenous TFs, but the remaining majority of cell lines still required the continuous expression of the exogenous genes to maintain their self-renewal. Gao et al. utilized the porcine TFs to produce the putative iPSCs followed by small molecular inhibitor screening and culture system modification with these iPSCs, and finally successfully established porcine expanded potential stem cells (EPSCs) from embryo (Gao et al., 2019). The main purpose of the researchers adopting the different species of TFs in the above studies was to harvest the high quality of iPSCs or to screen the small molecular, however the intrinsic mechanism of the activation of endogenous pluripotency markers had not been closely concerned. The amino acid sequence, the second structure and the tertiary structure of same protein in different species should be inconformity, the species distance determines the protein similarity. To check the structure similarity of the current used TFs among the different species origin and compare their activation efficiencies to endogenous TF will help illustrate the cell reprogram mechanism and establish the authentic naïve porcine iPSCs for application. In addition, as a closer species, cattle has not been used as TF origin for porcine PSCs’ induction.

In this study, we used pig, cattle and mouse derived TFs to reprogram the porcine fetal fibroblasts (PFFs) and compared the effects of different species origin of exogenous TFs on piPSCs generation. The results confirmed that the porcine TFs exhibited the highest level of activation effect of endogenous pluripotency markers and produced the optimal porcine iPSCs, better first than cattle and then mouse, which were compatible with the RNA sequencing results and the correlation results between the exogenous TFs expression and the endogenous counterparts activation in three kinds of piPSCs lines, as well as the bioinformation analysis results of protein characters of different species of TFs. Therefore, we concluded that the species affinity of the exogenous TFs is a key element, and the same species origin will be optimal for iPSCs generation and application. The porcine TFs derived piPSCs will become a useful material for pig gene editing and its successful establishment will provide an insight for the generation of porcine PSCs with germline transmission competency.

## 2 Materials and methods

### 2.1 Animal care and use

All experiments with animals were approved by the Institutional Animal Care and Use Committee (IACUC) of the Nanjing Medical University and the corresponding ethical approval code is IACUC-2112051. The methods were carried out in “accordance” with the approved guidelines.

### 2.2 Generation and culturation of piPSCs

The 4 main TFs OCT4, SOX2, KLF4, and CMYC from porcine, bovine and murine genome were used to reprogram Bama miniature PFFs and the derived piPSCs were named as ppiPSCs, bpiPSCs and mpiPSCs. The details were as follow.

To generate ppiPSCs and bpiPSCs, Bama miniature PFFs with a tdTomato cassette inserted into the 3′ UTR of the porcine OCT4 locus (POT PFFs) were transfected, which were kindly provided by Prof. Liangxue Lai (Guangzhou Institutes of Biomedicine and Health, Chinese Academy of Sciences) (Lai et al., 2016). The TFs combinations for ppiPSCs and bpiPSCs generation contained 2.0 μg PB-TRE-pOSKM (porcine OCT4, SOX2, KLF4 and CMYC) and 2.0 μg PB-TRE-bOSKM (bovine OCT4, SOX2, KLF4 and CMYC), respectively, and both also with 1.0 μg PB-TRE-pNhL (porcine NANOG and human LIN28), 1.0 μg PB-TRE-hRL (human RARG and LRH1), 1.0 μg PB-EF1a-transposase and 1.0 μg PB-EF1a-rTTA. The PB-TRE-bOSKM was constructed by Prof. Xihe Li’s group (Inner Mongolia University) (Zhao et al., 2021) and the remaining plasmids were kindly provided by Prof. Pentao Liu (University of Hong Kong) (Gao et al., 2019). After transfection, the cells and picked colonies were placed in culture dishes containing STO feeder layers and M15 medium (comprised knockout DMEM (Invitrogen, Carlsbad, CA, United States), 15% FBS, 1 × glutamine penicillin-streptomycin (Invitrogen), 1 × NEAA (Invitrogen) and 0.1 mM 2-mercaptoethanol (Invitrogen), 50 μg/mL Vitamin C (VC) and 10 ng/mL hLIF (cat# LIF1010, Millipore)) with 1 mg/mL doxycycline (DOX) (cat# 04–0,018 Stemgent). The ppiPSCs and bpiPSCs were maintained on STO feeder layers, enzymatically passaged every 3–5 days by a brief DPBS wash followed by treatment with TrypLE select (Gibco) for 3–5 min and incubated at 38.5°C in a 5% CO2 incubator.

For mpiPSCs generation, 8 μg DNA (4 μg TetO-FUW-mOSKM and 4 μg FUW-M2rtTA) was transferred into PFFs using a Lonza nuclear transfer machine under U-023. 24 h later, cells were passaged onto 10 cm dishes, and the same numbers of non-transgenic cells were used as negative control. On the next day, 300 ng/mL zeocin was applied to the culture medium. When the non-transgenic cells were completely drug-screened, the resistant cell colonies were digested in cloning cups and subcultured on mitotically inactivated mouse embryonic fibroblasts (MEFs) and induced by LBX medium. The mpiPSCs were enzymatically passaged and cultured with LBX medium when grown into a normal colony size. The LBX medium was supplemented with gene knockout serum replacement (KOSR) medium, N2B27 medium and supplemented with 16 ng/mL FGF (cat# 100-18B, PeproTech), 2000U/mL LIF and 2 mg/mL DOX. KOSR medium was knockout DMEM supplemented with 20% KSR (Invitrogen), 1% NEAA, 2 mM L-glutamine, 1% PS, 0.1 mM 2-mercaptoethanol, 3 mM CHIR99021 (cat# SML1046, Sigma), 1 mM PD0325901 (cat# PZ0162, Sigma), 2 mM SB431542 (cat# 1,614, Tocris) and 50 ng/mL VC. N2B27 medium was DMEM/F12 and Neurobasal supplemented with 1% N2 (cat# 17502048, Gibco), 2% B27 (cat# 17504044, Gibco), 0.25 mg/mL bovine serum albumin (BSA, cat# A4612, Sigma), 5 mg/mL insulin (Invitrogen), 1% PS, 3 mM CHIR99021, 1 mM PD0325901, 2 mM SB431542, and 50 ng/mL VC.

To obtain piPSCs that were independent of exogenous gene expression, we cultured the ppiPSCs and bpiPSCs in pEPSCs medium with minor modification (Gao et al., 2019), and mpiPSCs in dox-free LBX medium. The pEPSCs medium was N2B27 basal media (Knockout DMEM, 0.5% N2 supplement, 1% B27 supplement, 100 × glutamine penicillin-streptomycin, 100 × NEAA and 0.1 mM 2-mercaptoethanol) supplemented with 0.2 µM CHIR99021, 0.3 µM WH-4-023 (cat# 5413, Tocris), 2.5 µM XAV939 (cat# X3004, Sigma), 65.0 μg/mL VC, 10.0 ng mL hLIF, 20.0 ng/mL Activin A (cat# 120-14 E, PeproTech) and 0.3% FBS (BI, Kibbutz Beit-Haemek, Israel).

### 2.3 Analyses of integration of exogenous transcription factors

To assess the integration of foreign genes, the total genomes were extracted from different piPSCs cell lines harvested at passage 2. Total DNA was isolated from the cells for polymerase chain reaction (PCR) by the Genomic DNA Kit (cat# DP304-03, TIANGEN) according to the manufacturer’s instructions. DNA from PFFs was isolated as negative control and plasmids as positive control. The plasmid specific primers and specific porcine GAPDH primers were designed and used in PCR. The primer oligonucleotide sequences are shown in Supplementary Table S3.

### 2.4 Alkaline phosphatase staining and immunofluorescence for piPSCs

Alkaline phosphatase (AP) staining was performed with NBT/BCIP solution using a method provided by manufacturers (Promega, Madison, WI, United States). The cells were first washed with DPBS, and then fixed with 4% paraformaldehyde (Solarbio, Beijing, China) at room temperature for 10 min. Then, the cells were washed twice with DPBS and incubated with the reagent to avoid light for 15 min. After the staining was terminated with PBS, the cells were observed and photographed.

For immunofluorescence, cells were washed with DPBS and then fixed with 4% paraformaldehyde at room temperature for 10 min. Cells were permeabilized by 1% Triton X-100 (Sigma) for 10 min and blocked with 5% BSA at room temperature for 1 h. Then, the cells were incubated with the primary antibody overnight at 4°C, after three washes with DPBS, the cells were incubated with the secondary antibody for 1 h at room temperature. The nuclei were stained with DAPI (Sigma) and observed by fluorescence microscopy (Ti2, Nikon) and photographed. The primary antibodies were OCT4 (cat# sc5279, Santa), SOX2 (cat# 4900, Cell Signaling Technology), NANOG (cat# sc33759, Santa), βIII-tubulin (cat# ab68193, Abcam), Sma (cat# CBL171, Millipore), Keratin (cat# MAB1677, Millipore), LHX1 (cat# GTX129215, GeneTex), MIXL1 (cat# 22772-1-AP, proteintech), goat anti-rabbit IgG Alexa Fluor 546 (cat# AS007, ABclonal), goat anti-rabbit IgG Alexa Fluor 488 (cat# A11008, Thermo Fisher Scientific), goat anti-mouse IgG Alexa Fluor 546 (cat# A11003, Thermo Fisher Scientific), goat anti-mouse IgG Alexa Fluor 488 (cat# A32723, Thermo Fisher Scientific).

### 2.5 Karyotype analysis

The cells were cultured with stem cell culture medium containing 0.02 μg/mL colchicine (KaryoMAXTM Colcemid^™^ Solution in PBS) for 1–1.5 h at 38.5°C in an incubator of 5% CO_2_. After digestion and treatment with preheated hypotonic KCl (0.56%, Sigma) for 30min at 37°C, the cells were fixed in a 3:1 mixture of methanol and glacial acetic acid. Suspension was dropped onto clean, precooled slides. After air drying, the chromosomes were stained by Giemsa (1:10 dilution) for 20 min and observed and recorded at 1,000 magnification (80i, Nikon).

### 2.6 Quantitative Real-Time PCR analysis

To detect the expression of endogenous and exogenous pluripotency genes in the cells, piPSCs were harvested at passage 5. Total RNA was extracted using the Total RNA Kit (cat# LS1040, Promega) according to the manufacturer’s instructions and was reverse transcribed into cDNA using HiScriptII Q RT Super Mix (Vazyme, Nanjing, China). The cDNA obtained from these cell lines was used for Quantitative Real-Time PCR (qPCR) reactions. qPCR was performed using ChamQ SYBR qPCR Master Mix (Vazyme) on a 96-well optical reaction plate in 7,500 Real-time System (Applied Biosystems, Waltham, MA, United States). *GAPDH* was used as an internal control to standardize 2^−ΔΔCT^ values of target genes. Overall gene expression was assessed by normalizing the gene expression of the samples to that of parental embryonic porcine fibroblasts. Data were analyzed by Ct method and significance was determined at *p* ≤ 0.05. The primers used in this study are shown in Supplementary Table S3.

### 2.7 Embryoid body formation assay

For embryoid bodies (EBs) formation, piPSCs were cultured in hanging drops without bFGF, hLIF, and other small molecule inhibitors for 3 days. Then EBs were placed on a 24-well plate covered with gelatin and cultured in the same conditions for 7–10 days. The differentiation of the three germ layer cells was identified by RT-qPCR and immunofluorescence.

### 2.8 Differentiation of piPSCs to PGCLCs

To induce piPSCs differentiating into incipient mesodermal-like cells (iMeLCs), they were first cultured in 12-well plates (1.5–3.0 × 10^5^/well) coated with fibronectin for 2 days. The mpiPSCs, bpiPSCs and ppiPSCs were cultured in the medium containing 50 ng/mL Activin A, 3 μM CHIR99021 and 10 μM Y27632 (cat# 1,254, Tocris). Then, to differentiate cells to pPGCLCs, iMeLCs were digested into single cells and transferred into U-shaped 96 wells at 5 × 10^3^. They were induced for 6 days in basal medium supplemented with 200 ng/mL BMP4 (cat# 120–05, PeproTech), 10 ng/mL hLIF, 100 ng/mL SCF (cat# 300–07, PeproTech) and 50 ng/mL epidermal growth factor (EGF, cat# PHG0311, Invitrogen). Specific marker genes were detected by qPCR and immunofluorescence. 

### 2.9 Generation of chimera

The CAG-GFP plasmid was transfected into ppiPSCs using Nucleofector™ Kit 1 (cat# VPH-5012, Lonza). For chimeric contribution to mice, 10–15 GFP labeled ppiPSC-9 were injected into the middle post-posterior of E7.5 mouse embryos (ICR) and then cultured *in vitro* in human serum with KSOM and pEPSCs with DOX mixture medium (1:3) at 37°C in a 5% CO_2_ atmosphere for 12 h and 24 h for the evaluation of E8.5 chimerism.

### 2.10 RNA-seq library preparation and sequencing

A total amount of 3 mg RNA per sample from mpiPSC line 1 at passage 25, mpiPSC line 2 at passage 21, bpiPSC line 1 at passage 17, bpiPSC line 2 at passage 14, ppiPSC line 4 at passage 17 and ppiPSC line 7 at passage 19 were used as input materials for the RNA sample preparations. The libraries were constructed using TruSeq Stranded mRNA LT Sample Prep Kit (Illumina, San Diego, CA, United States) according to the manufacturer’s instructions. The transcriptome sequencing and analysis were conducted by OE Biotech Co., Ltd. (Shanghai, China). The libraries were sequenced on an Illumina HiSeq X Ten platform and 150 bp paired end reads were generated. *p*-value < 0.05 and foldchange >2 or foldchange < 0.5 was set as the threshold for significantly differential expression.

### 2.11 Statistical analysis

All data in this experiment were expressed as mean ± standard deviation (X ± S), and GraphPad software was used for significance analysis. *t*-test was used to compare the two independent samples and one-way ANOVA was used to compare multiple samples, and *p* ≤ 0.05 was considered statistically significant. In all graphs, *p* ≤ 0.05 was marked as *, *p* ≤ 0.01 was marked as **, *p* ≤ 0.001 was marked as ***, *p* ≤ 0.0001 was marked as ****, no statistical significance was marked as ns and n ≥ 3.

## 3 Results

### 3.1 The ppiPSCs exhibited the standard characterization of stem cells and initiated the endogenous expressions of *OCT4*, *SOX2*, *KLF4*, *NANOG*, *LIN28*, *LRH1* and *RARG* except for *CMYC*

**FIGURE 1:**
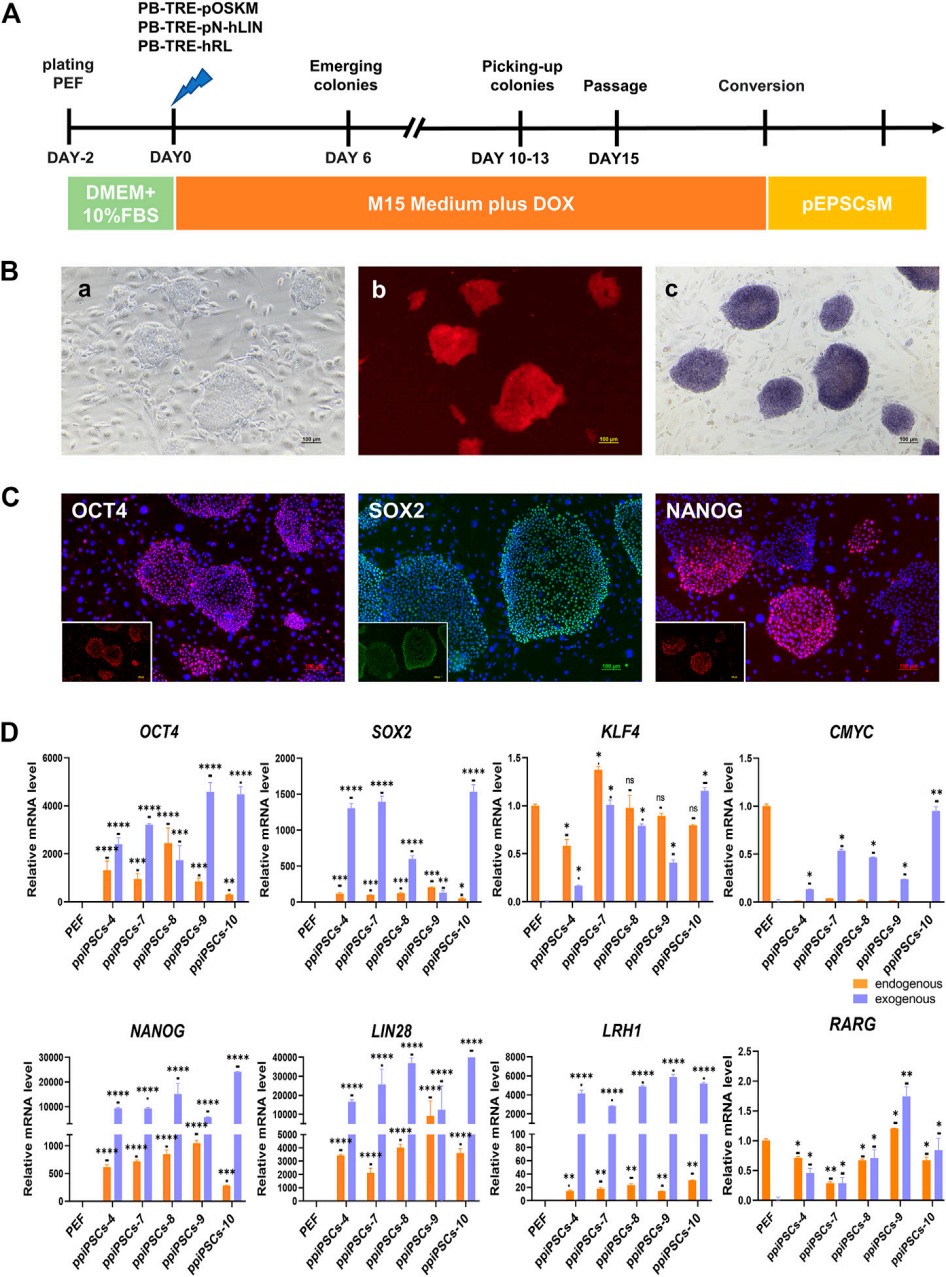
Derivation and characterization of ppiPSCs cells. **(A)** Schematic diagram of the establishment of ppiPSCs. **(B)** The morphology of ppiPSCs. **(a)** The morphology of ppiPSCs-4 colony at passages 21. Scale bars, 100 μm; **(b)**. PpiPSCs-4 cell line was OCT4-tdTomato^+^; **(c)**. AP staining of ppiPSCs cells. **(C)** The immunofluorescence staining of pluripotency markers OCT4, SOX2 and NANOG in ppiPSCs colonies cultured on STO cells. Scale bars, 100 mm. **(D)** qPCR analysis of endogenous and exogenous transcription factors of *OCT4*, *SOX*2, *KLF*4, *CMYC*, *NANOG*, *LIN28*, *LRH1* and *RARG* in ppiPSCs lines 4, 7, 8, 9 and 10. Data are depicted as mean ± SD. *p < 0.05, **p < 0.01, ***p < 0.001, ****p < 0.0001, ns, no statistical significance.

The POT PFFs were transfected with three DOX inducible piggyBac plasmids including pOSKM (porcine OCT4, SOX2, KLF4 and CMYC), pNhL (porcine NANOG and human LIN28) and hRL (human RARG and LRH1) to generate ppiPSCs (Figure 1A). The primary iPSC-like colonies were firstly observed on day 6 after DOX induction, which exhibited compact morphology and a high nuclei-to-cytoplasm ratio. These colonies were collected on day 12 and subcultured in single-cell suspension on STO feeder layers in serum-containing medium (M15 medium) plus DOX (Figure 1Ba). Total 15 ppiPSCs lines were obtained and all of them have 8 exogenous TFs integrated and stably expressed (Supplementary Figure S1A). All ppiPSCs cell lines were OCT4-tdTomato^+^ (Figure 1 Bb; Supplementary Figure S1B). The ppiPSCs could be passaged for more than 30 generations in single cells with no change in morphology and proliferation rate, and all were AP positive (Figure 1Bc). The core pluripotency markers such as OCT4, SOX2 and NANOG were positive by immunofluorescence staining (Figure 1C). Analysis of Quantitative Real-Time PCR (qPCR) showed that the 7 endogenous TFs *OCT4*, *SOX2*, *KLF4*, *NANOG*, *LIN28*, *LRH1* and *RARG* were activated and expressed except for *CMYC* in these cell lines (Figure 1D; Supplementary Figure S1C).

### 3.2 The bpiPSCs exhibited the characterization of stem cells and initiated the endogenous expression of *OCT4*, *SOX2*, *NANOG*, *LIN28*, *LRH1* and *RARG* except for *KLF4* and *CMYC*

The POT PFFs were transfected with three DOX inducible piggyBac plasmids including bOSKM, pNhL and hRL to generate bpiPSCs (Supplementary Figure S2A). The primary iPSC-like colonies were firstly observed on day 6 after DOX induction and colonies exhibiting compact and three-dimensional, well-defined colony morphology were picked on days 10–13 (Supplementary Figure S2B a). The bpiPSCs could be passaged in single-cell suspension and maintained in M15 medium plus DOX. Total 8 cell lines were obtained, which have 8 exogenous TFs integrated and stably expressed (Supplementary Figure S3A). The No. One bpiPSCs cell line (bpiPSCs-1) from POT PFFs were OCT4-tdTomato^+^ (Supplementary Figure S3B b), but the remaining 7 bpiPSCs cell lines were OCT4-tdTomato^-^ (Supplementary Figure S3B). The bpiPSCs showed AP positive (Supplementary Figure S2B c) as well as intranuclear expression of pluripotency markers including OCT4, SOX2 and NANOG (Supplementary Figure S2C). The qPCR confirmed the expression of eight exogenous TFs and the activation of porcine endogenous counterparts in bpiPSCs. The expression of the endogenous TFs *OCT4*, *SOX2*, *NANOG*, *LIN28*, *LRH1* and *RARG* were activated in the 8 cell lines although their expression patterns were different among individual cell lines (Supplementary Figure S2D; Supplementary Figure S3C). However, none bpiPSCs cell line initiated the endogenous expression of *KLF4* and *CMYC*. The eight bpiPSCs lines could be stably cultured *in vitro* for more than 35 passages and maintained normal karyotype.

### 3.3 The mpiPSCs exhibited the basic characterization of stem cells but only *SOX2* was endogenously activated

We transfected the expression vector TetO-FUW-mOSKM and the activation vector FUW-M2rtTA into PFFs, and mpiPSCs were generated through drug screening and DOX induction (Supplementary Figure S4A). The colonies could be observed on day 3 after induction and were picked on day 7. The mpiPSCs exhibited compact and dome-like morphology (Supplementary Figure S4B a) and were able to achieve single-cell colonization after enzymatic treatment. The mpiPSCs had normal karyotype after long-term culture (Supplementary Figure S4B b) and were AP positive (Supplementary Figure S4B c). The mpiPSCs expressed pluripotency markers, including OCT4, SOX2 and NANOG (Supplementary Figure S4C) with upregulated expression of four exogenous TFs. However, except for *SOX2*, the endogenous TFs *OCT4*, *KLF4* and *CMYC* remained barely expressed in the mpiPSCs (Supplementary Figure S4D). Ultimately, we obtained 2 cell lines that were able to continuously reproduce for more than 30 passages and maintain a robust self-renewal capacity.

### 3.4 The ppiPSCs exhibited the highest expression level of endogenous pluripotency genes compared with mpiPSCs and bpiPSCs

**FIGURE 2:**
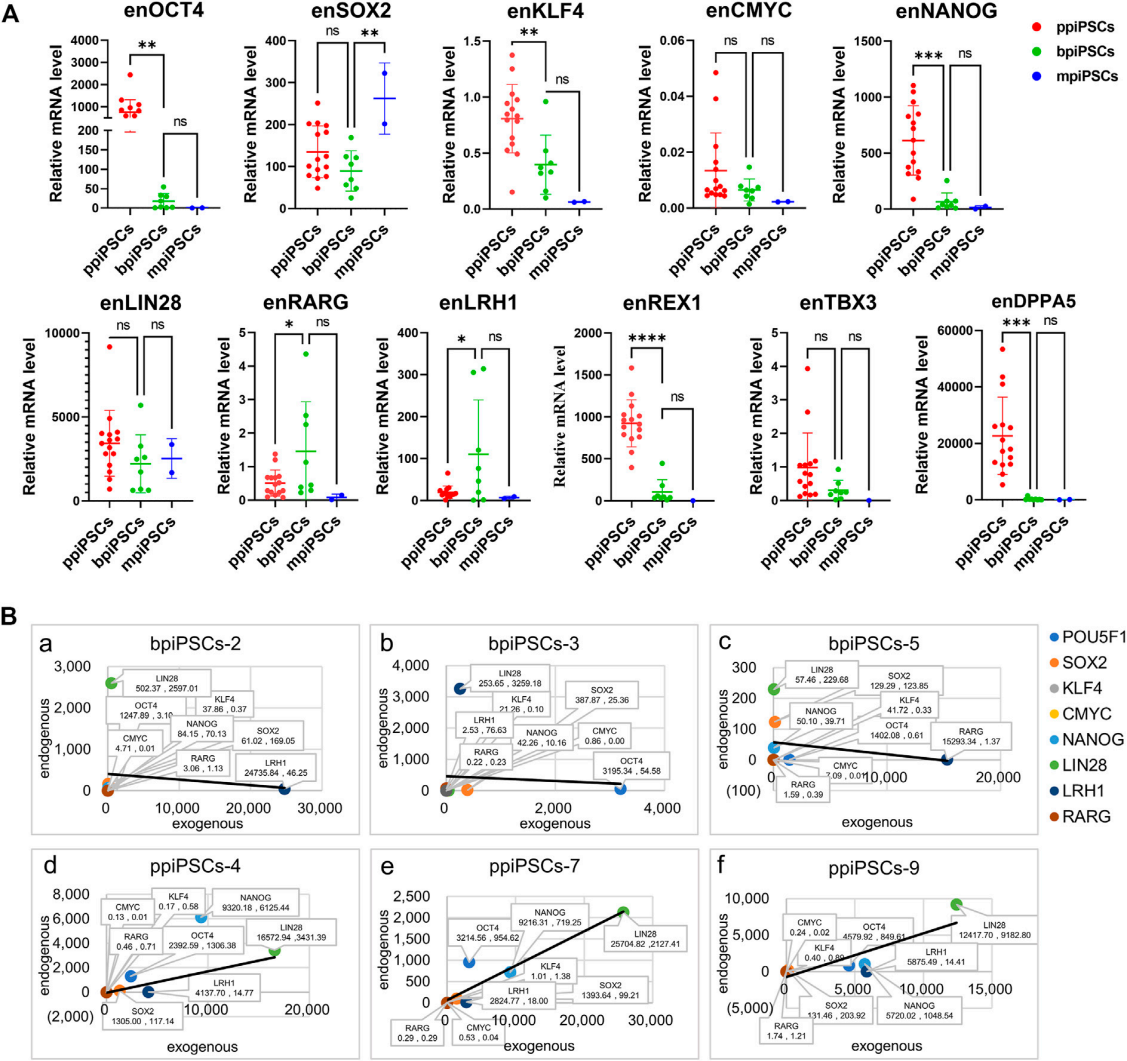
Comparison of endogenous pluripotency markers among the mpiPSCs, bpiPSCs and ppiPSCs and correlation analysis of endogenous and exogenous transcription factors. **(A)** qPCR analysis and comparison of endogenous pluripotency markers of *OCT4*, *SOX*2, *KLF*4, *CMYC*, *NANOG*, *LIN28*, *LRH1*, *RARG*, *TBX3*, *REX1* and *DPPA5* in ppiPSCs lines, bpiPSCs lines and mpiPSCs lines. Data are depicted as mean ± SD. *p < 0.05, **p < 0.01, ***p < 0.001, ****p < 0.0001, ns, no statistical significance. **(B)** Correlation analysis of the expression level between endogenous transcription factors and exogenous counterparts in piPSCs lines. a-c, bpiPSCs-2, 3 and 5; d-f, ppiPSCs-4, 7 and 9. The abscissa x represents the expression of exogenous transcription factors, and the ordinate y represents the endogenous counterparts. Data are presented in the form of (x, y).

We compared the effect of different species of exogenous TFs on the activation of endogenous pluripotency markers of three kinds piPSCs. The results showed that the average expression levels of the key endogenous pluripotency markers containing *OCT4*, *KLF4*, *CMYC*, *NANOG*, *REX1*, *TBX3*and *DPPA5* in ppiPSCs lines were the highest, and they were higher in bpiPSCs line and the lowest in mpiPSCs lines. The *LIN28* had the highest expression level in bpiPSCs and then mpiPSCs and ppiPSCs. In contrast, the *SOX2* had the highest expression level in mpiPSCs and then ppiPSCs and bpiPSCs. The *LRH1* and *RARG* had the highest expression level in bpiPSCs and then ppiPSCs and mpiPSCs (Figure 2A).

### 3.5 The expression level of the exogenous transcription factors and the endogenous counterparts indicated a positive correlation in ppiPSCs and a negative correlation in bpiPSCs

The correlation analysis of the expression level between 8 exogenous TFs and their endogenous counterparts showed the somewhat negative correlation in 62.5% (5/8) of the cell lines of bpiPSCs (Figure 2Ba-c; Supplementary Figure S5A). In contrast, 93.3% (14/15) of ppiPSCs showed a positive correlation, meaning that the activation of endogenous TFs enhanced when the expression level of exogenous counterparts increased. (Figure 2 B d-f; Supplementary Figure S5B).

### 3.6 The ppiPSCs had better *in vitro* differentiation potency, could integrate into E7.5 mouse embryo and proliferate independently of DOX

**FIGURE 3:**
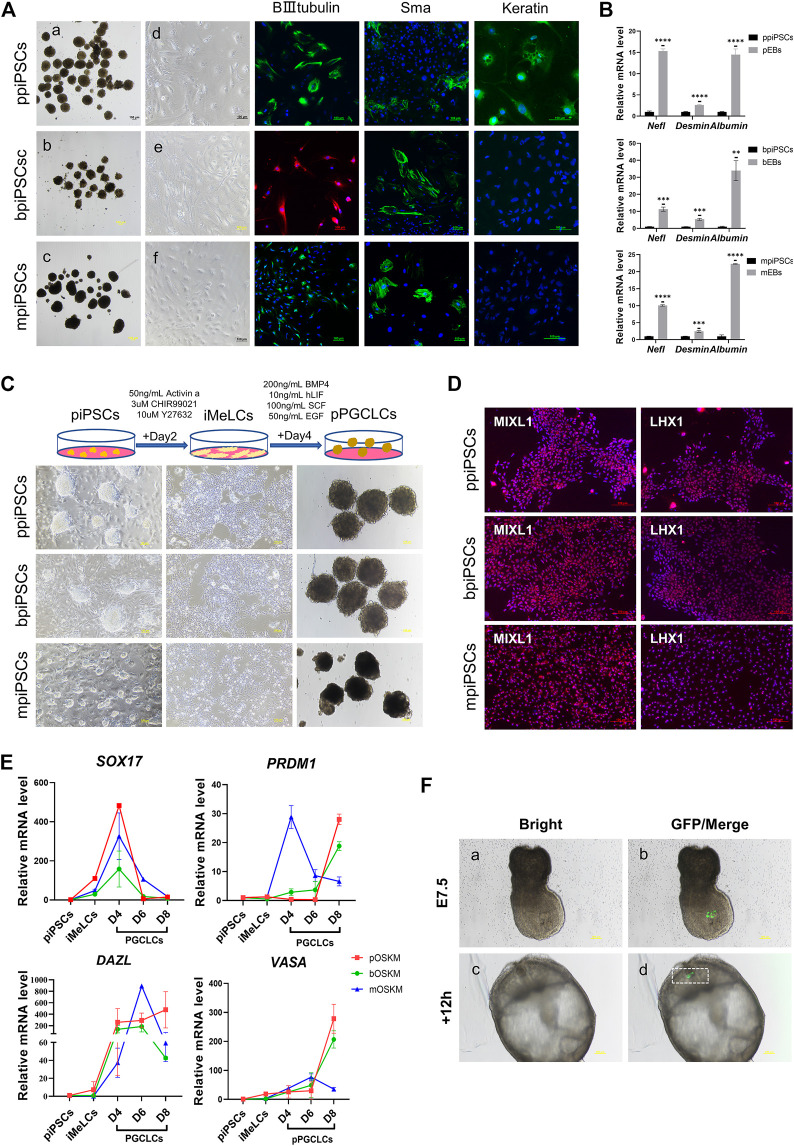
Differentiation potency of mpiPSCs, bpiPSCs and ppiPSCs *in vitro*. **(A)** a-c. EBs-like spheroids derived from mpiPSCs, bpiPSCs and ppiPSCs; d-f. EB-like spheroids spread on dishes coated with gelatin displayed distinct signs of differentiation. The immunofluorescence staining of differentiation markers βIII-Tubulin, Sma and Keratin in EBs from piPSCs. Scale bar, 100 µm. **(B)** qPCR analysis of the expression of differentiation-related genes in EB-like spheroids and piPSCs cells. Data are depicted as mean ± SD. *p < 0.05, **p < 0.01, ***p < 0.001, ****p < 0.0001, ns, no statistical significance. **(C)** Generation of pPGCLCs and their morphology. Scale bar, 100 µm. **(D)** Immunofluorescence analysis of mesodermal markers MIXL1 and LHX1. Scale bar, 100 µm. **(E)** qPCR analysis of the expression of key genes for PGC specification and development in piPSCs, iMeLCs and pPGCLCs. Data are depicted as mean ± SD. **(F)** Analysis of the contribution of ppiPSCs to E7.5 mouse embryos. **(a, b)** Injection of GFP labeled ppiPSCs-9 to E7.5 mouse embryos. **(c, d)** Injected E7.5 mouse embryos cultured for 12 h. Scale bar, 100 µm.

All three kinds of piPSCs could form EBs-like spheroids when grown in suspension for 7 days (Figure 3Aa-c). Day-4 EB were plated on gelatin-coated dishes under the same conditions, and the cells of EBs showed a markedly differentiated morphology on day 10(Figure 3 A d-f). The EBs from ppiPSCs (pEBs) were able to differentiate into three germ layers (ectoderm *ß*-III tubulin, mesoderm Sma and endoderm Keratin). EBs from bpiPSCs (bEBs) and mpiPSCs (mEBs) expressed *ß*-III tubulin and Sma but did not express Keratin (Figure 3A). The expressions of differentiation-related genes, such as ectoderm *Nefl*, mesoderm *Desmin* and endoderm *Albumin*, in EBs-like spheroids derived from all three kinds of piPSCs lines were significantly upregulated compared with undifferentiated counterparts (Figure 3B). In short, all piPSCs had the ability to differentiate into multiple germ layers. However, ppiPSCs exhibited the full-scale differentiation potential *in vitro* toward the three germ layers.

It has been reported that human PSCs were able to transform into PGCLCs via iMeLCs (Yamashiro et al., 2020). After optimizing the induction system, we developed a procedure to differentiate into PGCLCs from piPSCs (pPGCLCs) (Figure 3C). The ppiPSCs could rapidly and efficiently differentiate into PGCLCs (ppPGCLCs). However, only one of two bpiPSCs cell lines could differentiate toward PGCLCs (bpPGCLCs) and express few reproduction-related markers, but mpiPSCs only differentiated to the iMeLCs could not reach to PGCLCs. Compared to mpiPSCs differentiated cell mass (putative mpPGCLCs), ppPGCLCs and bpPGCLCs proliferated vigorously and both of ppPGCLCs and bpPGCLCs formed clusters with bird’s nest-like colonies, which are typical morphological features of PGCs *in vitro*. After 2 days of induction, the iMeLCs from three kind of piPSCs expressed mesodermal markers LHX1 and MIXL1 (Figure 3D). qPCR results indicated that the expression of mesodermal markers *MIXL1* and *GATA3* were significantly upregulated in all pPGCLCs but the expression in ppPGCLCs was at the highest level compare with bpPGCLCs and putative mpPGCLCs (Supplementary Figure S6A). We also analyzed the gene expression dynamics during 8 days of pPGCLCs induction (Figure 3E). During the formation of ppPGCLCs, the expression of the key genes for PGC specification and development were significantly upregulated, including *PRDM1*, *DAZL* and *VASA*. In bpPGCLCs, the expression of *PRDM1* and *VASA* gradually increased, whereas *DAZL* was significantly increased on day 4 and 6 but downregulated on day 8. For putative mpPGCLCs, *PRDM1* and *DAZL* reached peaks at D4 and D6 respectively and declined significantly thereafter. The expression of *SOX17* in all pPGCLCs showed exhibited a trend of high-level expression in the early stage and downregulation in the later stage, which was consistent with the results of PGC formation in mice (Hayashi et al., 2011).

To further investigate the developmental capacity of piPSCs, chimera experiments were performed. First, the ppiPSCs were transfected with the CAG-GFP plasmid (Supplementary Figure S6B). We then injected 10–15 GFP labeled ppiPSCs into the distal region of a late-streak stage embryo and allowed them to develop further for 12 and 24 h *in vitro*. GFP^+^ cells were detected in the host embryos (Figure 3F) and contributed to the neural ectoderm (Supplementary Figure S6C). Collectively, ppiPSCs could differentiate and contribute to chimeric formation in mouse late embryos.

**FIGURE 4:**
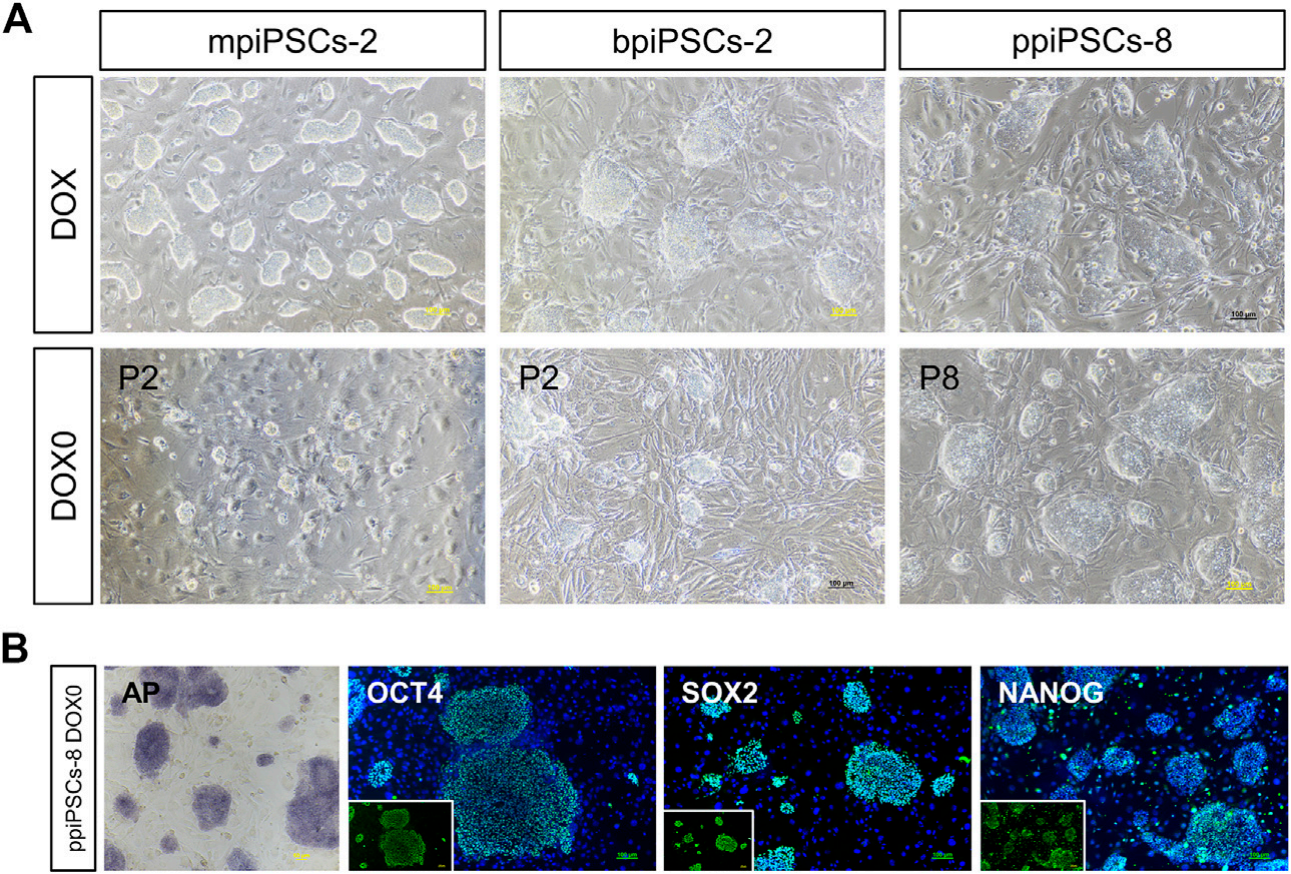
The morphology and pluripotency analysis of piPSCs after DOX withdrawal. **(A)** The morphology of piPSCs lines with or without DOX. Scale bar, 100 µm. **(B)** AP staining and immunofluorescence analysis of pluripotency markers OCT4, SOX2 and NANOG in ppiPSCs at passage 5 without DOX. Scale bar, 100 µm.

To obtain piPSCs that can proliferate independently of exogenous TFs expression, we cultured the piPSCs in DOX-free stem cell medium and observed the colony formation on the day 3. The mpiPSCs and bpiPSCs gradually died in 2 passages, whereas ppiPSCs showed no signs of altered morphology or differentiation and could be passaged normally (Figure 4A). The ppiPSCs were positive for AP staining and strongly expressed pluripotency markers including OCT4, SOX2, and NANOG at 5 passages without DOX (Figure 4B).

### 3.7 The ppiPSCs showed the distinct global gene expression profiling and the upregulated pluripotency signaling pathways compared with bpiPSCs and mpiPSCs

**FIGURE 5:**
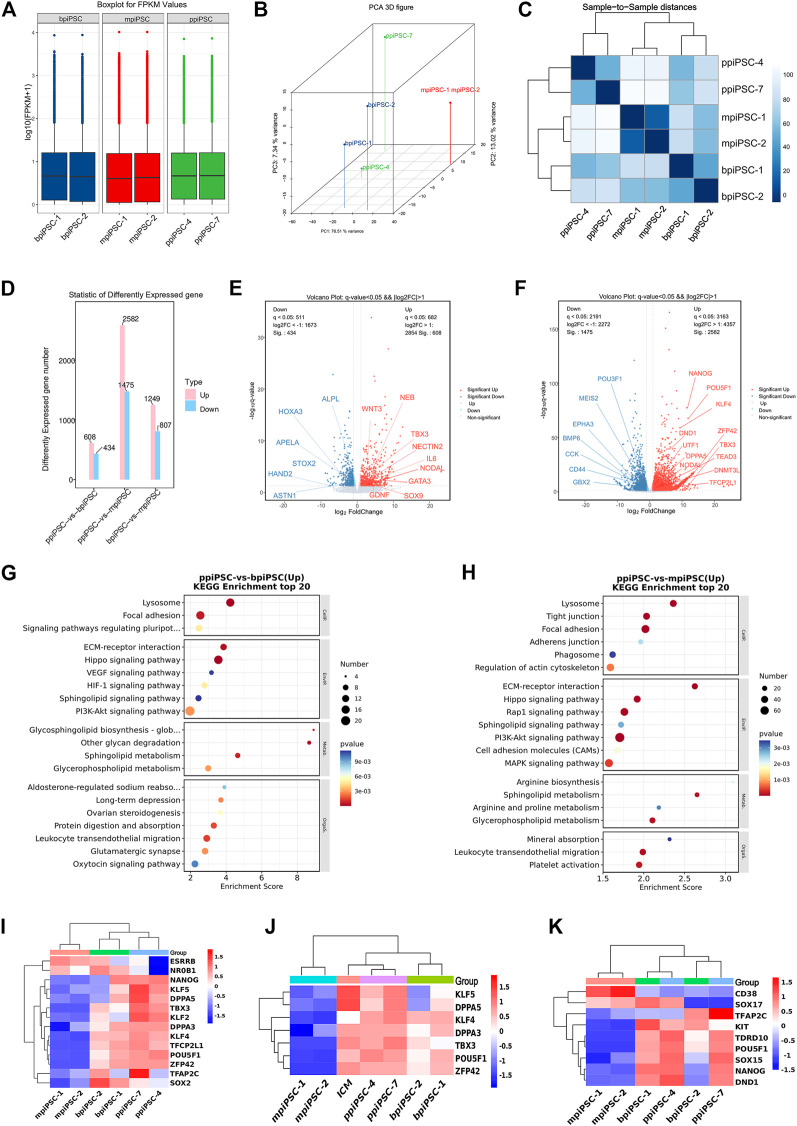
RNA-seq analysis of ppiPSCs, bpiPSCs and mpiPSCs. **(A)** Gene expression differences among mpiPSCs, bpiPSCs and ppiPSCs. **(B)** Global gene expression profiling of mpiPSCs, bpiPSCs and ppiPSCs. **(C)** Pearson correlation analysis of different kinds of cells. **(D–F)** Statistic and comparation of different expressed genes of mpiPSCs, bpiPSCs and ppiPSCs. **(G–H)** KEGG enrichment analysis of ppiPSCs and bpiPSCs and ppiPSCs and mpiPSCs. **(I)** Expression heatmap of endogenous pluripotency markers in mpiPSCs, bpiPSCs and ppiPSCs. **(J)** Expression heatmap of pluripotency genes for piPSCs compared with porcine ICM. **(K)** Expression heatmap of the PGC early related genes in mpiPSCs, bpiPSCs and ppiPSCs.

The RNA sequencing results indicated that ppiPSCs, bpiPSCs and mpiPSCs exhibited the obvious differences in gene expression pattern (Figure 5A). Global gene expression profiling revealed that gene expression in ppiPSCs was distinct from that in mpiPSCs and bpiPSCs (Figure 5B). Pearson correlation analysis confirmed that mpiPSCs and bpiPSCs show a stronger correlation in three kinds of piPSCs (Figure 5C). Compared with bpiPSCs, ppiPSCs had 608 upregulated genes (such as *TBX3*, *NODAL*, *SOX9*) and 434 downregulated genes (such as *HAND2*, *HOXA3*, *APELA*) among the 15,473 identified genes (Figures 5D,E). KEGG enrichment analysis showed that the upregulated genes were mostly enriched in Hippo signaling pathway, PI3K-Akt signaling pathway and the other signaling pathways regulating pluripotency of stem cells (Figure 5G). Compared with mpiPSCs, ppiPSCs had 2,582 upregulated genes (such as *POU5F1*, *NANOG*, *KLF4*, *DPPA5*, *TBX3*) and 1,475 downregulated genes (such as *BMP6*, *CCK*, *POU3F1*) among the 15,432 identified genes (Figures 5D,F). KEGG enrichment analysis showed that the upregulated genes were mostly enriched in PI3K-Akt signaling pathway, Hippo signaling pathway and MAPK signaling pathway (Figure 5H). We selected genes related to pluripotency from RNA-seq data for analysis and confirmed that ppiPSCs expressed most endogenous pluripotency markers, and bpiPSCs expressed a few endogenous pluripotency markers, but mpiPSCs lack the expression of most endogenous pluripotency markers (Figure 5I). We also confirmed that ppiPSCs robustly express *POU5F1*, *KLF4*, *DPPA5*, *TBX3*, *ZFP42* and other genes related to pluripotency. This expression pattern was consistent with that of porcine ICM (RNA-seq data obtained previously by our lab) (Zhang et al., 2019), which also highly express these genes (Figure 5J). In addition, we chose the PGC early related genes for analysis, and found that both ppiPSCs cell lines strongly expressed most of these genes, a cell line of bpiPSCs highly expressed these genes, and mpiPSCs barely expressed these genes (Figure 5K).

### 3.8 The pig shares more similar amino acid sequences and protein structures in OCT4, SOX2 and KLF4 to cattle than to mouse

The similarities and differences of amino acid sequence, protein secondary structure and tertiary structure of the four key TFs OCT4, SOX2, KLF4 and CMYC in mouse, cattle and pig were compared *via* biological information analysis. Firstly, DNAMAN was used to compare the amino acid sequences, and the results showed that the similarity of OCT4 was 83% between pig and mouse and 97% between pig and cattle; the similarity of SOX2 was 97% among mouse, cattle and pig; of KLF4 was 92% between pig and mouse and 98% for pig and cattle; the similarity of CMYC was 92% for pig and mouse and 96% between pig and cattle (Supplementary Table S1). Secondly, Protean was used to compare protein secondary structure including *a*-helix, *ß*-fold and *ß*-turn amount. The total number of *a*-helix, *ß*-fold and *ß*-turn in OCT4 is 90.9, 91.9 and 88 for pig, cattle and mouse, respectively. They are 78.63, 78.07 and 82.09 in SOX2; 83, 83.93 and 83.67 in KLF4; 87.83, 86.78 and 92.1 in CMYC (Supplementary Table S2). These results revealed OCT4, SOX2, KLF4 and CMYC had more similar amino acid sequence and protein secondary structure between pig and cattle than pig and mouse. Finally, we used the online website SWISS MODEL to perform tertiary modeling and compared the tertiary structures using pyMOL. The RMSD values of mouse and pig at OSKM were 0.004, 0.003, 0.000, and 0.051, respectively and the values were 0.001, 0.003, 0.000, and 0.058 for cattle and pig, respectively (Supplementary Figure S6D). In conclusion, the protein characters of OCT4, SOX2 and KLF4 in pig were closer to cattle than to mouse. CMYC had more similar tertiary structure between pig and mouse.

## 4 Discussion

The core TFs determining the pluripotency and self-renewal of stem cells have been well investigated (Kashyap et al., 2009). *OCT4* has been identified to play a major role in mouse, human and rat iPSCs production and serves as a major pluripotency marker (Kashyap et al., 2009). *NANOG* was reported to be another key factor for the pluripotent stem cell induction from bovine adult fibroblasts (Sumer et al., 2011) and *LIN28* was essential for maintaining pluripotency in porcine iPSCs (Chakritbudsabong et al., 2021). *TBX3* and *LRH1* were identified to play important roles in establishing mESCs-like porcine iPSCs (Wang et al., 2013). *REX1* is an important gene associated with naïve status (Nichols and Smith, 2009). In this study, we pay more attention to investigate the activation of the endogenous pluripotency markers of piPSCs, and we found that ppiPSCs expressed more kinds and the highest level of the endogenous pluripotency markers, but bpiPSCs expressed a few endogenous pluripotency markers, the mpiPSCs even lack the expression of the most of endogenous pluripotency markers. The endogenous expression level of *OCT4* was sequentially reduced in porcine, bovine and murine TFs derived piPSCs. All the ppiPSCs lines showed red fluorescence *via* expressing the endogenous *OCT4*-tdtomato knocked-in in the initial cells, but only one bpiPSCs line was *OCT4*-tdtomato positive. These results suggest that *OCT4* is the most critical factor for piPSCs pluripotency maintenance and self-renewal. We also surprisingly found that the other core TFs *CMYC*, *NANOG*, *LIN28*, *DPPA5*, *TBX3* and *REX1* had the same expression pattern as *OCT4*, with the highest levels of expression in ppiPSCs, and then in bpiPSCs and mpiPSCs. The expression level of endogenous pluripotency factors in ppiPSCs is consistent with the previously reported expression of ICM in porcine embryos (Zhang et al., 2019).

The complete reprogramming of the cell requires the sufficient activation of endogenous TFs, while silencing of the exogenous counterparts to stably maintain the pluripotent state (Maherali et al., 2007; Wernig et al., 2007). Since the incomplete silencing of exogenous transgenes affect iPSCs differentiation potential and its further application, elimination of transgene expression became one of the most important steps (Okita et al., 2007; Okada and Yoneda, 2011). Self-renewal dependent on exogenous TF expression has been a major problem in large animal iPSCs, including porcine iPSCs (Esteban et al., 2009; Wu et al., 2009; Zhang et al., 2014; Du et al., 2015). In our study, mpiPSCs and bpiPSCs showed a dependence on Dox-induced transgene expression, and differentiated spontaneously after Dox withdrawing, suggesting that mpiPSCs and bpiPSCs are in a pre-iPSC state. Notably, ppiPSCs stably survived and strongly expressed pluripotency markers after dox withdrawal, indicating that the cell lines can overcome the barriers throughout the reprogramming process, such as initiation, maturation and stabilization. The endogenous activation of individual TF seems not to be an independent event. The positive correlation between the expression level of the exogenous transcription factors and the endogenous counterparts in ppiPSCs and a negative correlation in bpiPSCs, indicated the interactivities of all the inducing TFs and the interactions of the exogenous TFs and the endogenous TFs, thus there might be the complicated gene network or signaling cascade during the endogenous TFs activation. Based on the above analysis, in contrast to mpiPSCs and bpiPSCs, the most important reason for the survival of ppiPSCs should be attributed to the sufficient activation of the endogenous TFs and the interactivities of all the involved TFs. The complete reprogramming of the cell also requires the accurate global transcriptional profiling. Hippo signaling pathway is thought important in modulating proliferation or differentiation of stem cells (Varelas, 2014; Chung et al., 2016) and controlling lineage differentiation of mESCs through modulating the super-enhancers formation (Sun et al., 2020). The PIK-Akt pathway was shown in mESCs to activate Nanog to regulate the core pluripotency (Niwa et al., 2009). The MAPK signaling pathway is directedly related to the pluripotency maintenance of porcine iPSCs (Wu et al., 2021). The porcine TFs derived piPSCs presented the homogeneity between the individual cell lines with the three signaling pathways upregulated, which further confirmed the significance of the global gene networks.

It is important to reconstruct germ cell development *in vitro* for PSCs. The great progresses have been made in the induction of PGCLCs from mouse and human PSCs (Hayashi et al., 2011; Sasaki et al., 2015). The mouse epiblast-like PSCs had been directly induced into PGCLCs, and even developed normally functioning gametes *in vitro* (Hayashi et al., 2011). Human iPSCs were differentiated into iMeLCs first and then PGCLCs (Sasaki et al., 2015). Most importantly, human PGCLCs eventually were differentiated into oogonia/gonocyte-like cells (Yamashiro et al., 2018), demonstrating their germline competency. We also generated porcine PGCLCs from ppiPSCs, bpiPSCs and mpiPSCs. The three kinds of pPGCLCs exhibited quite different morphology, the ppPGCLCs and bpPGCLCs had typical bird’s nest-like colony, consistent with the previous reported cell clusters of PGCs *in vitro*, whereas mpPGCLCs exhibited irregular contour in aggregates. At the iMeLCs state, ppiPSCs acquired more sufficient expression levels of *MIXL1* and *GATA3* and eventually developed to PGCLCs. The ppiPSCs derived PGCLCs highly expressed PGC markers *PRDM1*, *DAZL* and *VASA*, and the expression patterns were similar to that of germ cells, suggesting that ppiPSCs could differentiate into germline cells more rapidly and efficiently. The heterogeneity in gene expression profiles of mpPGCLCs may negatively affect their differentiation potential.

In this study, we used the exact same induction system for derivation of ppiPSCs and bpiPSCs, Although the induction system employed in mpiPSCs is different in the numbers of TFs and culture media, it contained the same 4 core TFs. In addition, all three kinds of piPSCs were reprogrammed from Bama miniature PFFs. So the different characters of ppiPSCs, bpiPSCs and mpiPSCs could be mainly ascribed to TFs’ species origin. The results that the different animal species derived TFs can inordinately re-program porcine somatic cells, suggesting that pluripotency-related signaling networks are conserved across species. Based on the similarity analysis of protein structures and the results of endogenous initiation levels of pluripotency markers, we suggest that using of TFs with high similarity to those reprogrammed cells (preferably of the same species) can better activate the expression of endogenous counterparts in PSCs. This study will provide insights for the research of large animal PSCs, and the established ppiPSCs will become experimental materials for biomedicine, regenerative medicine and reproductive medicine.

## Data Availability

The datasets presented in this study can be found in online repositories. The names of the repository/repositories and accession number(s) can be found below: NCBI BioProject with accession number, PRJNA915487.
