# Supplementary material for: Species origin of exogenous transcription factors affects the activation of endogenous pluripotency markers and signaling pathways of porcine induced pluripotent stem cells
Source: Front Cell Dev Biol. 2023 Apr 21;11:1196273. doi: 10.3389/fcell.2023.1196273 (PMC10160484; doi:10.3389/fcell.2023.1196273)
Supplement: Supplementary file 1 [file Table1.DOCX]

Supplementary Material

# Supplementary Tables

**Supplementary Table 1** Amino acid sequence alignment of key transcription factors OCT4, SOX2, KLF4 and CMYC among mouse, cattle and pig

| **Identify** | **OCT4** | **SOX2** | **KLF4** | **CMYC** |
| --- | --- | --- | --- | --- |
| Mouse/Pig | 83% | 97% | 92% | 92% |
| Cattle/Pig | 97% | 98% | 98% | 96% |

**Supplementary Table 2** Protein secondary structure alignment of key transcription factors OCT4, SOX2, KLF4 and CMYC among mouse, cattle and pig

| **proportion** | **OCT4** | | | **SOX2** | | | **KLF4** | | | **CMYC** | | |
| --- | --- | --- | --- | --- | --- | --- | --- | --- | --- | --- | --- | --- |
|  | Mouse | Pig | Cattle | Mouse | Pig | Cattle | Mouse | Pig | Cattle | Mouse | Pig | Cattle |
| α-helix | 30.4 | 33.6 | 34.4 | 28.8 | 21.9 | 22.5 | 26.1 | 27.4 | 27.3 | 38.3 | 37.4 | 36 |
| β-sheet | 28.1 | 21.7 | 21.9 | 4.39 | 5.33 | 4.37 | 7.87 | 7 | 7.13 | 12.8 | 9.73 | 8.88 |
| β-turn | 29.5 | 35.6 | 35.6 | 48.9 | 51.4 | 51.2 | 49.7 | 48.6 | 49.5 | 41 | 40.7 | 41.9 |
|  | 88 | 90.9 | 91.9 | 82.09 | 78.63 | 78.07 | 83.67 | 83 | 83.93 | 92.1 | 87.83 | 86.78 |

**Supplementary Table 3.** The primer sequence of Real-time PCR.

| **Gene** | **Sequence（5′-3′)** | | **Length（bp）** |
| --- | --- | --- | --- |
| pOSKM | Forward | ACGACTCGGTGCAGCCTTAT | 2706 |
|  | Reverse | GCCCAGTATGTTTGCGGTAG |  |
| bOSKM | Forward | GCTCTGGTAGTGCTGGGACA | 1360 |
|  | Reverse | GAGAAAGCGGACGAGTATCG |  |
| pN-hLIN | Forward | CCAATGCCTGAGGTTTATG | 1726 |
|  | Reverse | TCAGGTAGCCACCTACTGCT |  |
| hRL | Forward | GGCTGTGGGACAAGTTCAGT | 1269 |
|  | Reverse | TGGTCAGGTCAGAGGGCATA |  |
| enOCT4 | Forward | AGCAGTGACTATTCGCAACG | 302 |
|  | Reverse | CCAAAGCCCTGGTACAAAC |  |
| enSOX2 | Forward | GTGAGGGCCGGACAGTGAACTG | 109 |
|  | Reverse | AAGCGTACCGGGTTTTTCTCCATAC |  |
| enKLF4 | Forward | GAACTGACCAGGCACTACCG | 258 |
|  | Reverse | GACTTGTTGGGAACTTGACC |  |
| enCMYC | Forward | TTACAACACCCGAGCGACAA | 153 |
|  | Reverse | CCACTATCCGAAGGAAATCCAG |  |
| enNANOG | Forward | CTGGAGGAGATCTTCATGATTCTAAG | 56 |
|  | Reverse | AGTCCAGGCTTAAGTGTCTAGATAGAAG |  |
| enLIN28 | Forward | GTTCGGCTTCCTGTCCAT | 124 |
|  | Reverse | CACAGCCTCACCCTCCTT |  |
| enRARG | Forward | AAGAGGTGAAGGAAGAAGGGTC | 138 |
|  | Reverse | TGGAGTTCGTGGTGTATTTGC |  |
| enLRH1 | Forward | GAAAGGGATTGGTGGTGA | 203 |
|  | Reverse | GCAATACAAATACCCTGATACA |  |
| enREX1 | Forward | GTCCTGAGAGTGGATGCACAAG | 179 |
|  | Reverse | CTGTGAACGGAGAGATGCTTTCT |  |
| enTBX3 | Forward | TGACGGCATACCAGAATGATAAGA | 212 |
|  | Reverse | CTGGGCAAAGCAGTTGAAGG |  |
| enDPPA5 | Forward | GATCTCGAATCCCGTATGT | 104 |
|  | Reverse | CAAGTAAGGACCGTAAACCA |  |
| m-exOCT4 | Forward | AGAAGTGGGTGGAGGAAGC | 207 |
|  | Reverse | ACCACATCCTTCTCTAGCCC |  |
| m-exSOX2 | Forward | TGGAGTGGGAGGAAGAGGTAA | 297 |
|  | Reverse | TGAACGGCTGGAGCAACG |  |
| m-exKLF4 | Forward | AGAAGGTCGTGGCCCCGGAA | 133 |
|  | Reverse | TCACAGTGGTAAGGTTTCTC |  |
| m-exCMYC | Forward | CCCTACCCGCTCAACGACAG | 169 |
|  | Reverse | GAGTCGCTGCTGGTGGTGGG |  |
| b-exOCT4 | Forward | AGAGGCAACCTGGAGAGCA | 222 |
|  | Reverse | CGCCAGAGGAAAGGATACG |  |
| b-exSOX2 | Forward | CATGCACCGCTACGACGTGAG | 202 |
|  | Reverse | CGCCCTGGAGTGGGAAGAAG |  |
| b-exKLF4 | Forward | CAGGTCCCACCGCTCCATT | 311 |
|  | Reverse | TCGCACTTCTGGCACTGGA |  |
| b-exCMYC | Forward | GAATGACAAGAGGCGGACAC | 132 |
|  | Reverse | AGGATAACTACCTTGGGGGC |  |
| p-exOCT4 | Forward | GTCGCCAGAAGGGCAAAC | 211 |
|  | Reverse | GTGACAGACACCGAGGGAAA |  |
| p-exSOX2 | Forward | AGCAAGCAGGTGATGTTGAAGAAA | 175 |
|  | Reverse | CGCTTAACTCGGTCTGGGCTGT |  |
| p-exKLF4 | Forward | AGCCCATCGGTCATCAGT | 267 |
|  | Reverse | TAGCAGTTCCTCCGCACCC |  |
| p-exCMYC | Forward | GGAGGAGAATGACAAGAGGC | 269 |
|  | Reverse | GTGGGCAAGAGTTCCGTAG |  |
| exNANOG | Forward | CTGTCTCTCCTCTTCCTTCC | 112 |
|  | Reverse | TTCTCTGTGCTCTTCTCTGC |  |
| exLIN28 | Forward | CCCCAGTGGATGTCTTTGTG | 108 |
|  | Reverse | GACCCTTGGCTGACTTCTTA |  |
| exRARG | Forward | AGCGTTGTCCTTACTGTCGTT | 251 |
|  | Reverse | GGCAGAGTGGATGTTTTGAAT |  |
| exLRH1 | Forward | TACACCCCAGAGCAGGACAC | 114 |
|  | Reverse | CCCAGCAAAGGCAAAGACAA |  |
| NEFL | Forward | AAACGCCGCTATGTGGAGAC | 163 |
|  | Reverse | CGAGACTGGGCATCAAGGAG |  |
| DESMIN | Forward | TGTCCAAGCCAGACCTCACC | 249 |
|  | Reverse | GCCTCATCAGGGAATCGTTAG |  |
| ALBUMIN | Forward | GCGCTCATAGTTCGTTACAC | 157 |
|  | Reverse | TCAGGACCAGGGACAGATAG |  |
| GATA3 | Forward | AACCACGTCCCGTCCTACTA | 199 |
|  | Reverse | GGTGGATGGACGTCTTGGAG |  |
| SOX17 | Forward | CTGGAGGAGCGGAGCAAATC | 173 |
|  | Reverse | CAGGGCAACTGTGGGAAACC |  |
| PRDM1 | Forward | CAGTGCCGTGAAGTTTCCA | 189 |
|  | Reverse | AAGGATGCCTCTGCCTGAAC |  |
| DAZL | Forward | GGGTCGCTTTGCTTATCCGC | 183 |
|  | Reverse | TGCAGCAGACATTACTGCGA |  |
| VASA | Forward | CCTGCCCAGGAATGCCATCA | 179 |
|  | Reverse | ACTGGCCAACTTGGAGAATGGT |  |

pOSKM/bOSKM: porcine or bovine derived transcription factor OCT4, SOX2, KLF4 and CMYC; pN-hLIN: porcine NANOG and human LIN28; hRL: human RARG and LRH1; en-: endogenous; m-ex-: murine derived transcription factor; b-ex-: bovine derived transcription factor; p-ex-: porcine derived transcription factor; ex-: exogenous.
