# Supplementary material for: Species origin of exogenous transcription factors affects the activation of endogenous pluripotency markers and signaling pathways of porcine induced pluripotent stem cells
Source: Front Cell Dev Biol. 2023 Apr 21;11:1196273. doi: 10.3389/fcell.2023.1196273 (PMC10160484; doi:10.3389/fcell.2023.1196273)
Supplement: Supplementary file 2 [file DataSheet1.pdf]

Supplementary Material

1 Supplementary Figures

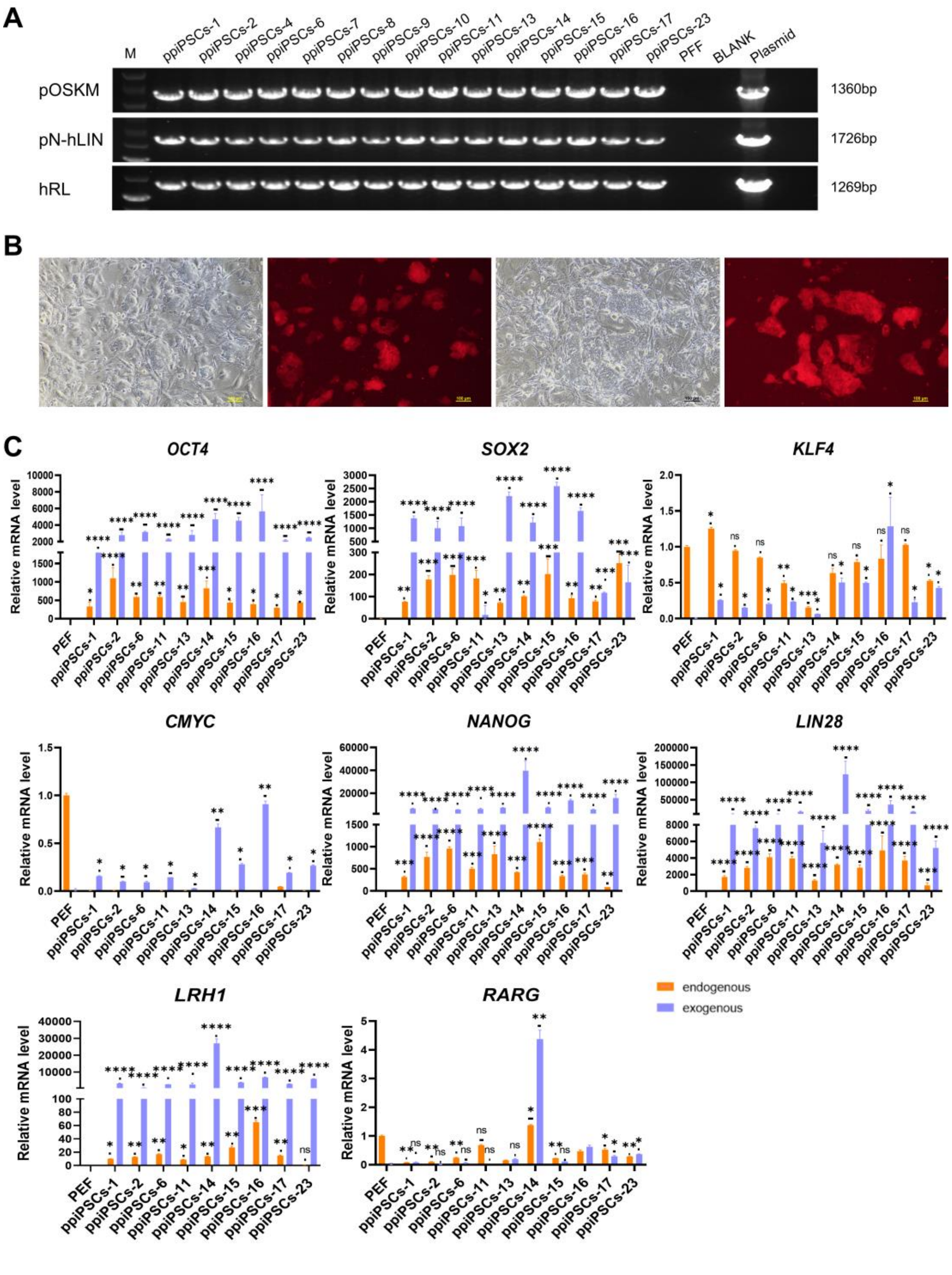

Supplementary Figure 1. Characterization of ppiPSCs lines. (A) RT-PCR analysis of exogenous transcription factors integration of ppiPSCs cells. (B) The ppiPSCs-10 and ppiPSCs-8 cell lines

exhibited OCT4-tdTomato+. Scale bars, 100 µm. (C) qPCR analysis of endogenous and exogenous transcription factors of *OCT4*, *SOX2*, *KLF4*, *CMYC*, *NANOG*, *LIN28*, *LRH1* and *RARG* in ppiPSCs lines 1, 2, 6, 11, 13, 14, 15, 16, 17 and 23. Data are depicted as mean ± SD. * $p<0.05$, ** $p<0.01$, *** $p<0.001$, **** $p<0.0001$, ns, no statistical significance.

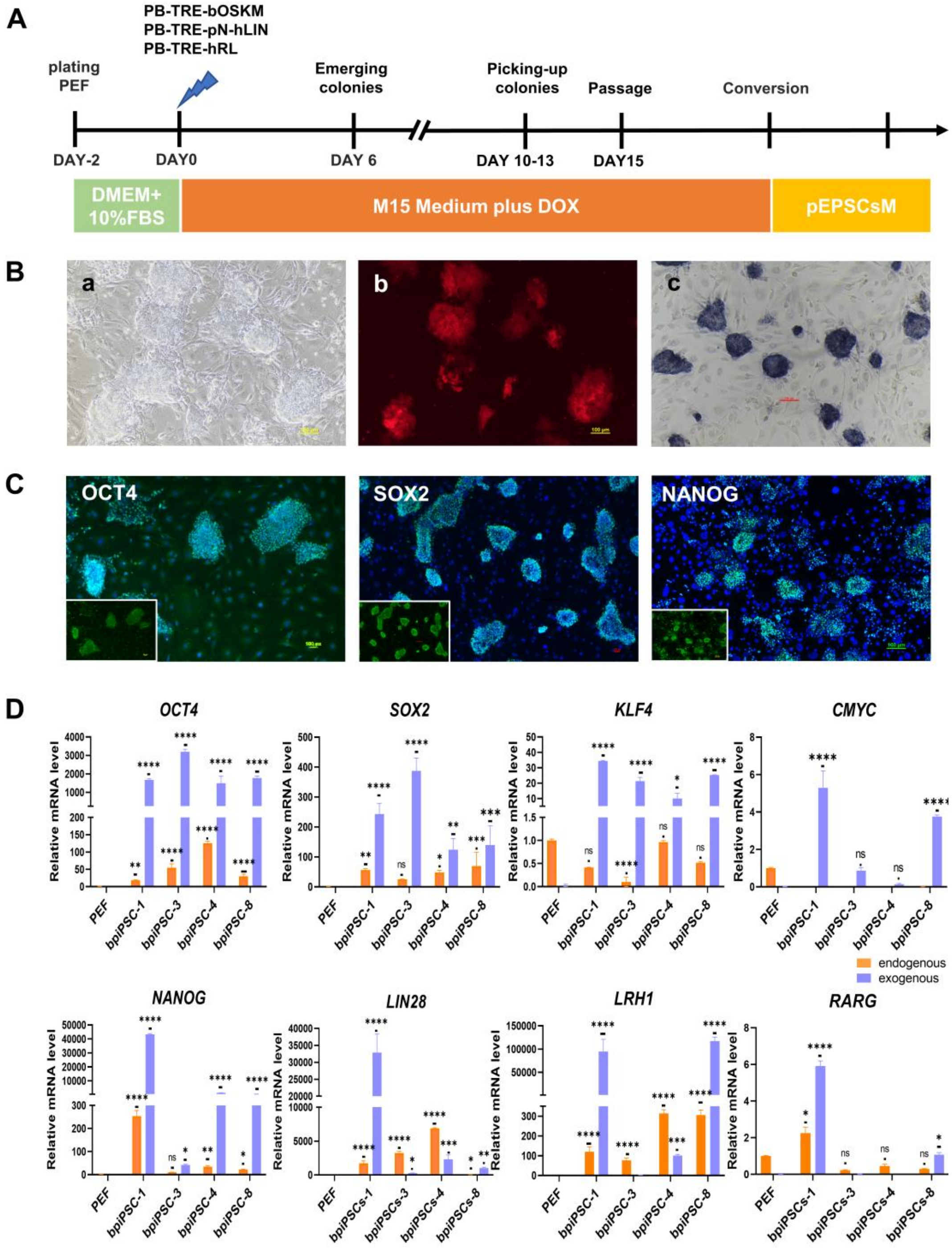

Supplementary Figure 2. Derivation and characterization of bpiPSCs cells. (A) Schematic diagram of the establishment of bpiPSCs. (B) a. The morphology of bpiPSCs-1 colony at passages 21. Scale bars, 100 µm; b. ppiPSCs-1 cell lines was OCT4-tdTomato+; c. AP staining of bpiPSCs cells. (C) The immunofluorescence staining of pluripotency markers OCT4, SOX2 and NANOG in bpiPSCs

colonies cultured on STO cells. Scale bars, 100 µm. (D) qPCR analysis of endogenous and exogenous transcription factors of *OCT4*, *SOX2*, *KLF4*, *CMYC*, *NANOG*, *LIN28*, *LRH1* and *RARG* in ppiPSCs lines 1, 3, 4 and 8. Data are depicted as mean ± SD. * p<0.05, ** p<0.01, *** p<0.001, **** p<0.0001, ns, no statistical significance.

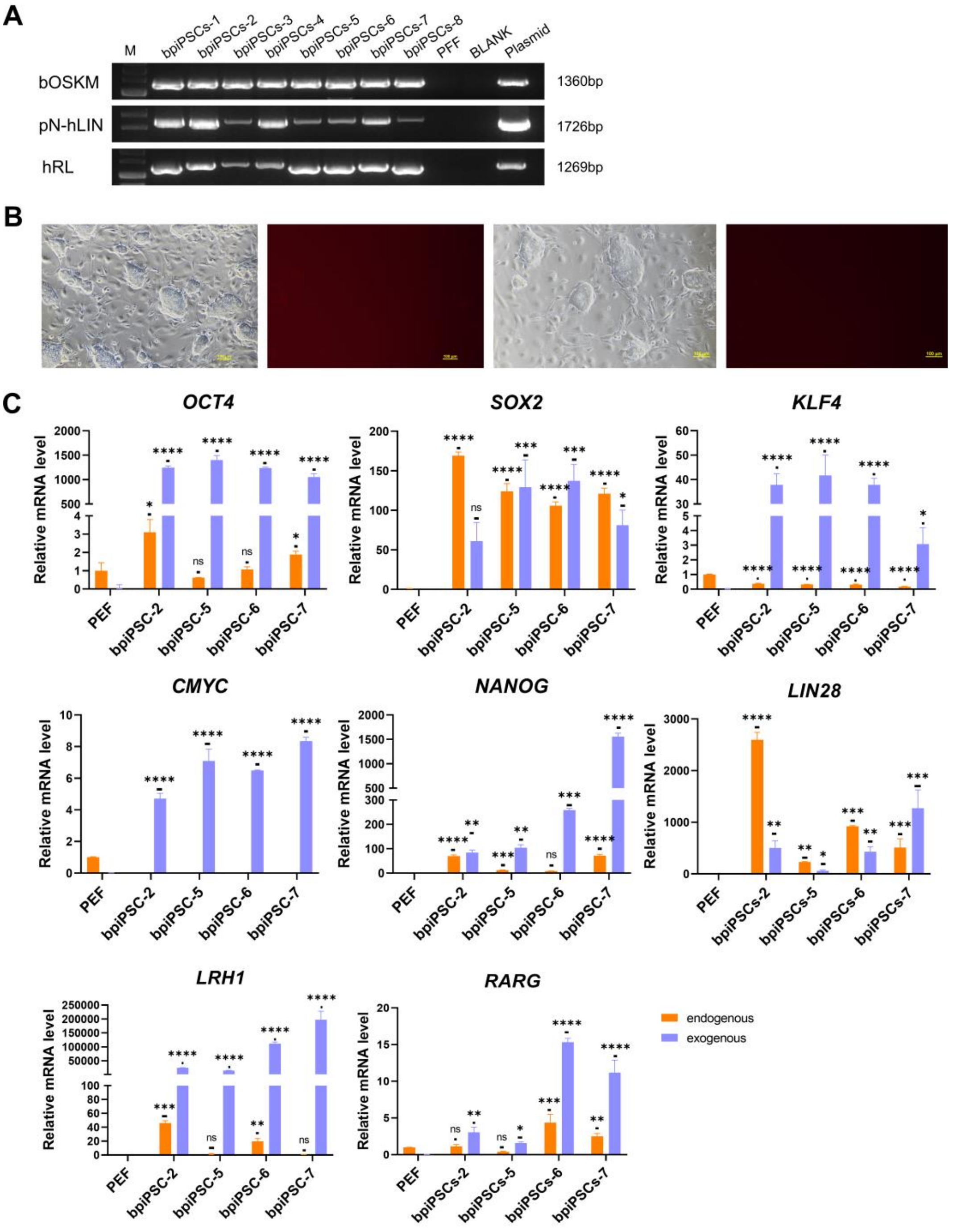

Supplementary Figure 3. Characterization of bpiPSCs lines. (A) RT-PCR analysis of exogenous transcription factors integration of bpiPSCs cells. (B) The bpiPSCs-2 and bpiPSCs-3 exhibited OCT4-tdTomato-. Scale bars, 100 µm.(C) qPCR analysis of endogenous and exogenous transcription factors of OCT4, SOX2, KLF4, CMYC, NANOG, LIN28, LRH1 and RARG in bpiPSCs lines 2, 5, 6

and 7. Data are depicted as mean ± SD. * p<0.05, ** p<0.01, *** p<0.001, **** p<0.0001, ns, no statistical significance.

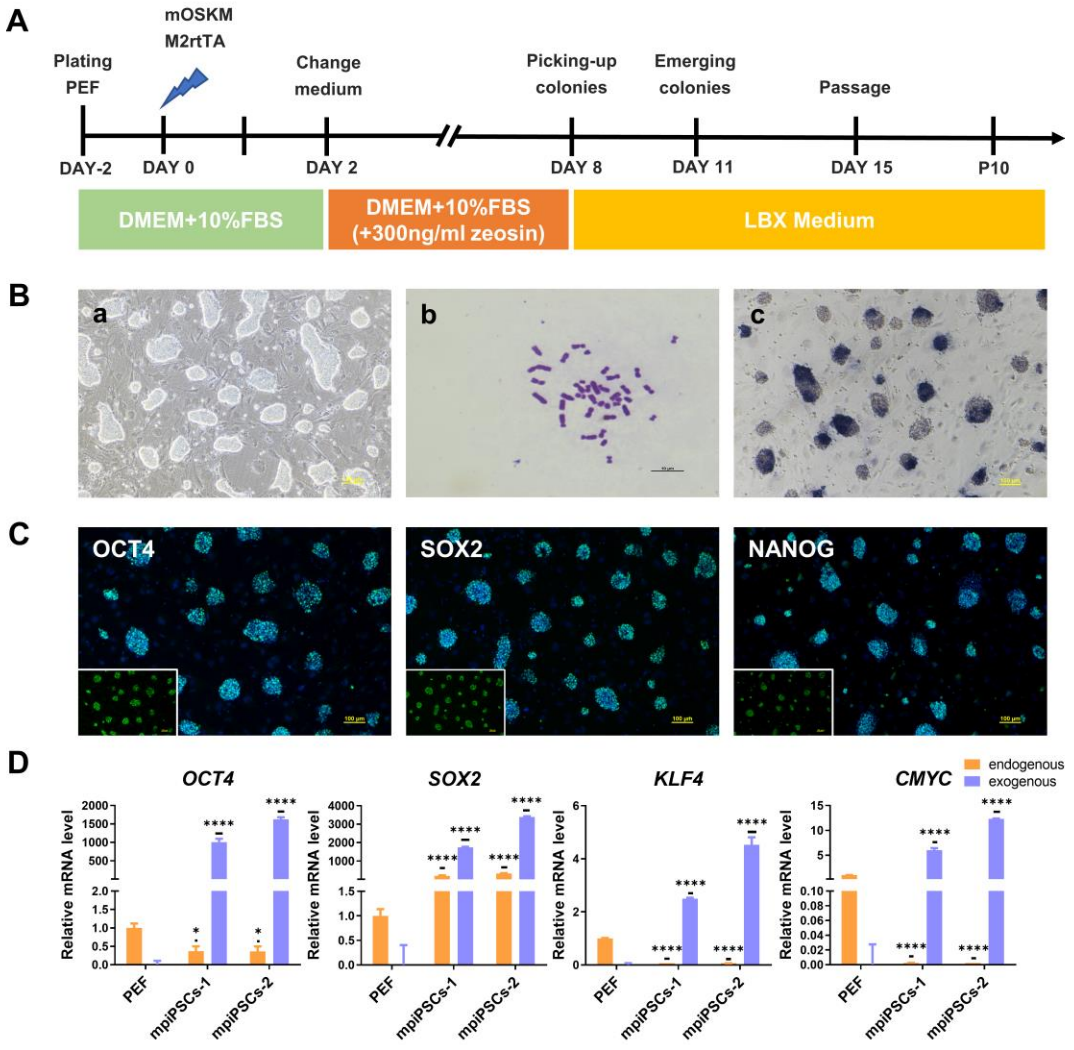

Supplementary Figure 4. Derivation and characterization of mpiPSCs cells. (A) Schematic diagram of the establishment of mpiPSCs. (B) a. The morphology of mpiPSCs-2 colony at passages 31. Scale bars, 100 µm; b. Karyotype analysis of mpiPSCs line 2 at passage 25; c. AP staining of mpiPSCs cells at passage 17. (C) The immunofluorescence staining of pluripotency markers OCT4, SOX2 and NANOG in mpiPSCs colonies (at passage 17) cultured on MEF cells. Scale bars, 100 µm. (D) qPCR analysis of the expression of endogenous and exogenous transcription factors *OCT4*, *SOX2*, *KLF4* and *CMYC* of in mpiPSCs lines 1 and 2. Data are depicted as mean ± SD. * p<0.05, ** p<0.01, *** p<0.001, **** p<0.0001, ns, no statistical significance.

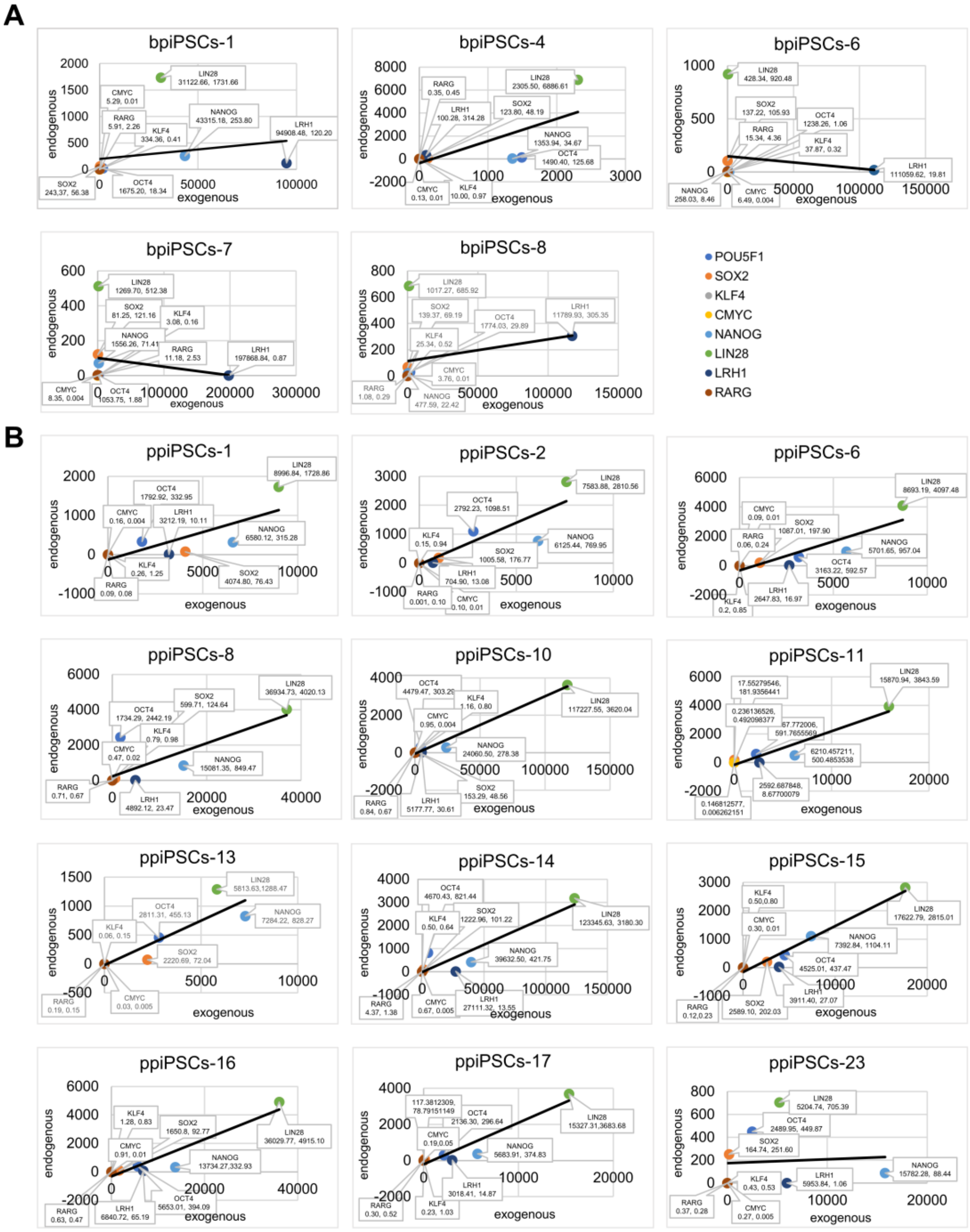

Supplementary Figure 5. Correlation analysis of the expression level between endogenous and exogenous transcription factors in bpiPSCs and ppiPSCs lines. (A) Correlation analysis in bpiPSCs1, 4, 6, 7 and 8. (B) Correlation analysis of in ppiPSCs1, 2, 6, 8, 10, 11, 13, 14, 15, 16, 17 and 23. The abscissa X represents the expression of exogenous transcription factors, and the ordinate Y represents the endogenous counterparts. Data are presented in the form of (X, Y).

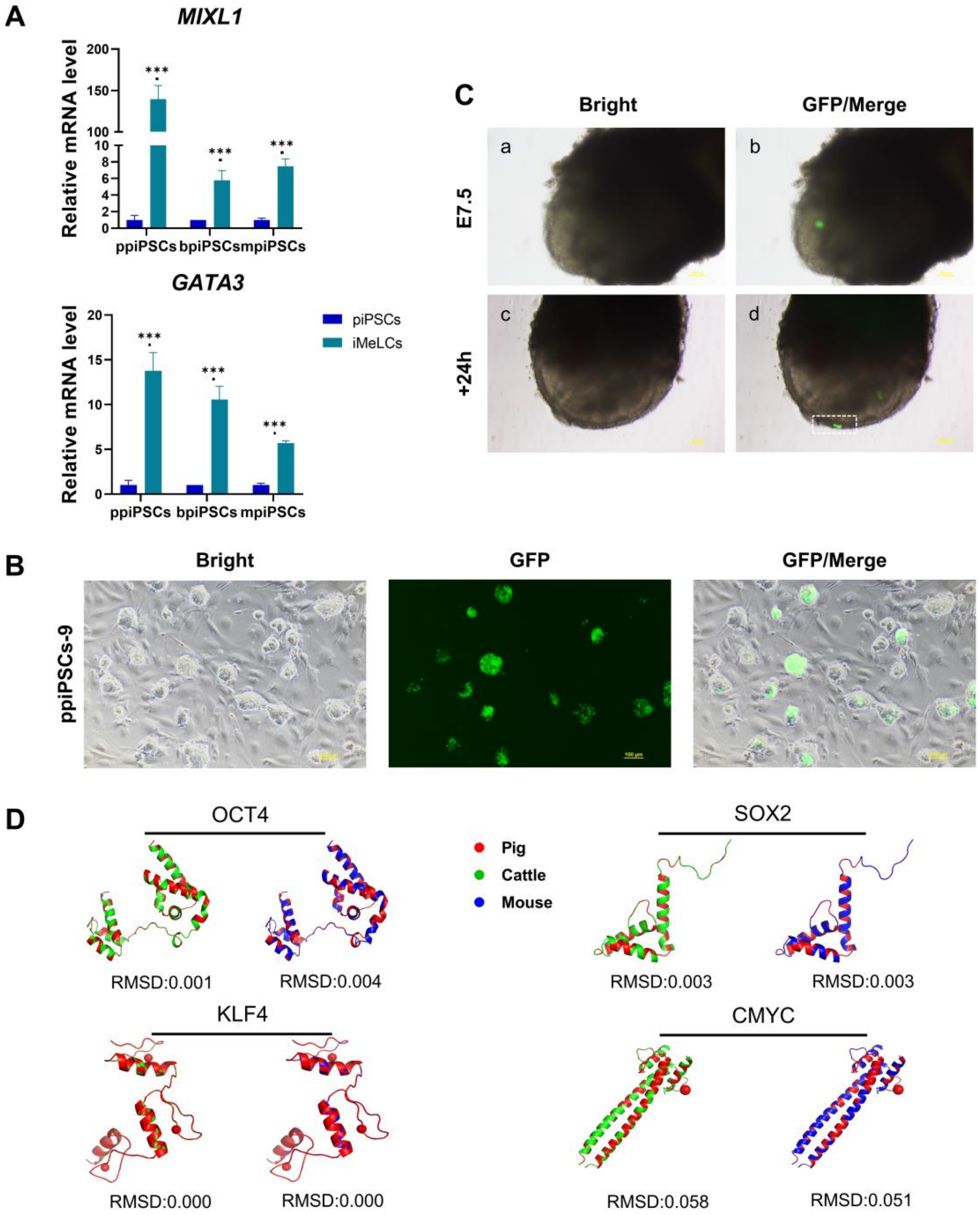

Supplementary Figure 6. The differentiation potency of piPSCs and the similarities of tertiary structure of the key transcription factors in different species. (A) qPCR for the expression of incipient mesoderm related genes in piPSCs cells and iMeLCs. Data are depicted as mean ± SD. *p<0.05, **p<0.01, ***p<0.001, ****p<0.0001. (B) Labeling ppiPSCs-9 with GFP. Scale bar 100 μm. (C) Analysis of the contribution of ppiPSCs-9 to E7.5 mouse embryos. (a, b) Injection of GFP labeled ppiPSCs-9 to E7.5 mouse embryos. (c, d) Injected E7.5 mouse embryos cultured for 24 h. Scale bar, 100 μm. (D) Similarities of tertiary structure of the key transcription factors OCT4, SOX2, KLF4 and CMYC in mouse, cattle and pig.
